# Molecular Characterization of a Novel Germline* VHL* Mutation by Extensive* In Silico* Analysis in an Indian Family with Von Hippel-Lindau Disease

**DOI:** 10.1155/2016/9872594

**Published:** 2016-03-16

**Authors:** Gautham Arunachal, Divya Pachat, C. George Priya Doss, Sumita Danda, Rekha Pai, Andrew Ebenazer

**Affiliations:** ^1^Department of Clinical Genetics, Christian Medical College, Vellore, Tamil Nadu 632004, India; ^2^School of Biosciences and Technology, VIT University, Vellore, Tamil Nadu 632014, India; ^3^Department of Pathology, Christian Medical College, Vellore, Tamil Nadu 632004, India

## Abstract

Von Hippel-Lindau [VHL] disease, an autosomal dominant hereditary cancer syndrome, is well known for its complex genotype-phenotype correlations. We looked for germline mutations in the* VHL* gene in an affected multiplex family with Type 1 VHL disease. Real-Time quantitative PCR for deletions and Sanger sequencing of coding regions along with flanking intronic regions were performed in two affected individuals and one related individual. Direct sequencing identified a novel heterozygous single nucleotide base substitution in both the affected members tested, segregating with VHL phenotype in this family. This variant in exon 3, c.473T>A, results in substitution of leucine, a highly conserved acid, to glutamine at position 158 [p.L158Q] and has not been reported thus far as a variant associated with disease causation. Further, this variant was not observed in 50 age and ethnicity matched healthy individuals. Extensive* in silico* prediction analysis along with molecular dynamics simulation revealed significant deleterious nature of the substitution L158Q on pVHL. The results of this study when collated support the view that the missense variation p.L158Q in the Elongin C binding domain of pVHL may be disease causing.

## 1. Introduction

Von Hippel-Lindau [VHL] syndrome is an autosomal dominantly inherited cancer syndrome with an incidence of 1 in 36,000 live births [1]. VHL syndrome is characterized by multiple tumor types affecting different organ systems with an age dependent penetrance pattern. Among the most frequent tumors are hemangioblastomas [HB] of the CNS and retina, clear cell carcinoma of kidneys [RCC], pheochromocytoma, endolymphatic sac tumors [ELSTs], pancreatic neuroendocrine tumors [NETs], and paragangliomas. Also, cysts of the pancreas, renal, epididymal, and broad ligament are seen in varying proportions.

The disease not only is known for its phenotypic heterogeneity, but also demonstrates significant interfamilial and intrafamilial variations which are partly explained by well known complex genotype-phenotype correlations.* VHL*, a tumor suppressor, is the only gene in which heterozygous germline mutations are known to cause VHL disease [2]. The evidence of biallelic inactivation of* VHL* found in tumor samples is consistent with Knudson's “two-hit model” where a mutation in one of the alleles is inherited, and the wild-type allele is somatically inactivated leading to tumorigenesis.

All types of mutations including deletions have been described in the literature. Over 900 nonrecurrent mutations have been described so far, although mutational hot spots and some founder mutations in certain ethnic groups have also been observed [2]. With the exception of deletions which account for 30–40% of cases, most mutations cluster in two regions of high functional importance in VHL protein [pVHL], the Elongin C binding site [amino acid residues 157–171] and HIF1 alpha [amino acid residues 91–113], which are highly evolutionarily conserved regions [3, 4].

Considering the occurrence of phenotypic heterogeneity and interfamilial variations in VHL, historically it has been classified into five disease phenotypes based on the probability of consistent occurrence of either pheochromocytoma or RCC in the kindred [5]. VHL Type 1 families are characterized by HB with RCC and are thought to have a low risk of pheochromocytoma. It is further subtyped as Type 1a in those families with predisposition to both HB and RCC and Type 1b in those families with predisposition predominantly to HB with low risk of RCC. The loss of function mutations due to either truncating mutations or exonic deletions is often associated with Type 1 VHL phenotype [5]. VHL Type IIa is associated with high risk of pheochromocytoma and HB as well as low risk of renal cell carcinoma while Type IIb is associated with high risk of pheochromocytoma, renal cell carcinoma along with HB. Type IIc VHL disease is characterized by occurrence of only pheochromocytoma in the particular kindred. Often missense mutations are associated with Type 2 VHL phenotype which accounts for around 10–20% of VHL kindreds [6, 7]. There are exceptions to these generalizations and in recent periods enough evidence exists to suggest that the families move from one class to another making the classification less meaningful clinically.

Genetic testing is indicated in all individuals suspected to have VHL syndrome along with the family members at risk [3]. Predictive testing of asymptomatic individuals and prenatal diagnosis have become possible because of increasing availability of molecular genetic testing. Here we describe the genetic workup of a family with multiple affected members with VHL and its role in genetic counseling. In addition, we employed a set of* in silico* prediction methods along with molecular dynamics simulation analysis to investigate whether the novel germline variant is disease causing or neutral.

## 2. Methods

### 2.1. Case Summary

**Figure 1:**
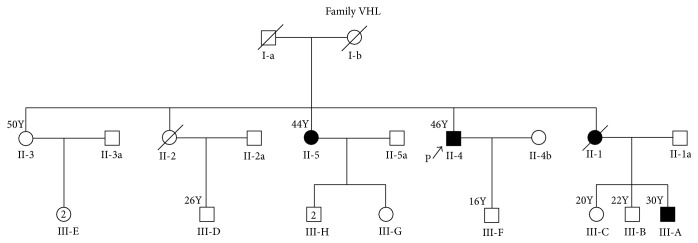
Pedigree of the affected family.

Proband, a 45-year-old man, born of nonconsanguineous parentage, presented to the Medical Genetics Clinic with history of bilateral retinal angiomas, multifocal cerebellar hemangioblastomas, and bilateral RCC. Most of the tumors had been promptly treated and at the time of presentation, he was on a recommended surveillance program. Upon further intensive investigations, there were no evidences for pheochromocytoma or ELSTs and pancreatic NETs. Family history revealed multiple first- and second-degree relatives affected with similar illness. Detailed pedigree is as shown in Figure 1. Of the four siblings, two were deceased. Among the two surviving, one is affected and another yet unaffected. Parents of the proband were deceased; the cause for their demise is unknown. With this history a diagnosis of Type 1 VHL was made and genetic testing was carried out for proband [II-4 in the pedigree], another affected relative [III-A, in the pedigree], and one yet unaffected relative [III-F, in the pedigree] after appropriate counseling and consenting process.

### 2.2. Molecular Genetic Testing

Peripheral blood samples were collected from two individuals affected with VHL, proband (II-4 in the pedigree) and his nephew (III-A in the pedigree), after appropriate consenting process (Figure 1). Genomic DNA was extracted from blood samples using NucleoSpin® Kit method (Macherey-Nagel, Germany). DNA extracted was quantified using Nano-Drop ND-1000 spectrophotometer [Thermo Fisher Scientific Commercial Services] and was subjected to polymerase chain reaction [PCR]. PCR was performed using appropriate primer sets and PCR conditions for exons 1, 2, and 3 covering the flanking intronic sequences as described previously [6]. Sequencing was performed using ABI 3130 genetic analyzer. Real-Time quantitative PCR [Applied 97500] was done as described previously [8], to check for large deletions/duplications which has been used reliably across many studies [9].

The above described method was employed after an appropriate consenting process to detect the presence or absence of the variants in the proband's son who is yet unaffected at the age of 18 years. Direct sequencing of 50 age and ethnicity matched controls was performed to detect the presence or absence of the novel variant found in the proband after receiving approval by the Institutional Review Board [IRB], Christian Medical College, Vellore. For the variants found, appropriate literature search, database search like dbSNP, HGMD, and extensive* in silico* prediction analysis were performed as necessary.

### 2.3. *In Silico* Prediction Analysis

To discriminate the impact of the new variant as disease causing or neutral, we employed various* in silico* prediction tools such as Sorting Intolerant from Tolerant (SIFT) [10], Polymorphism Phenotyping 2 (PolyPhen 2) [11], Screening for Non-Acceptable Polymorphisms (SNAP) [12], PROVEAN (Protein Variation Effect Analyzer) [13], MUpro [14], iStable [15] MuStab [16], and I-Mutant Suite [17]. These methods utilize sequence homology (conservation), amino acid physicochemical and structural properties to predict variant's effect on protein function and protein stability and pathogenicity. We submitted NCBI GI number, wild-type protein FASTA sequence, and wild and new residue after mutation (single-letter amino acid code) as input data for making their predictions.

In addition, we used Project Have yOur Protein Explained (HOPE) [18], which is an automatic mutant web server, to analyze the effect of a certain mutation on the protein structure.

### 2.4. ConSurf

To investigate the extent of conservation of amino acids, we used MUSCLE (Multiple Sequence Comparison by Log-Expectation), a web based tool to align multiple sequences from several vertebrate species including humans [19]. ConSurf utilizes Bayesian analysis to evaluate the evolutionary significance of the missense variant involved in a protein sequence [20]. We submitted the 3D structure with PDB ID as input to measure the functionally conserved residues in pVHL. ConSurf combines evolutionary and structural attributes such as solvent accessibility to make predictions. The scores represent extent of conservation with three categories: variable (1–4), intermediate (5-6), and conserved (7–9), respectively.

### 2.5. Molecular Dynamics

**Figure 2:**
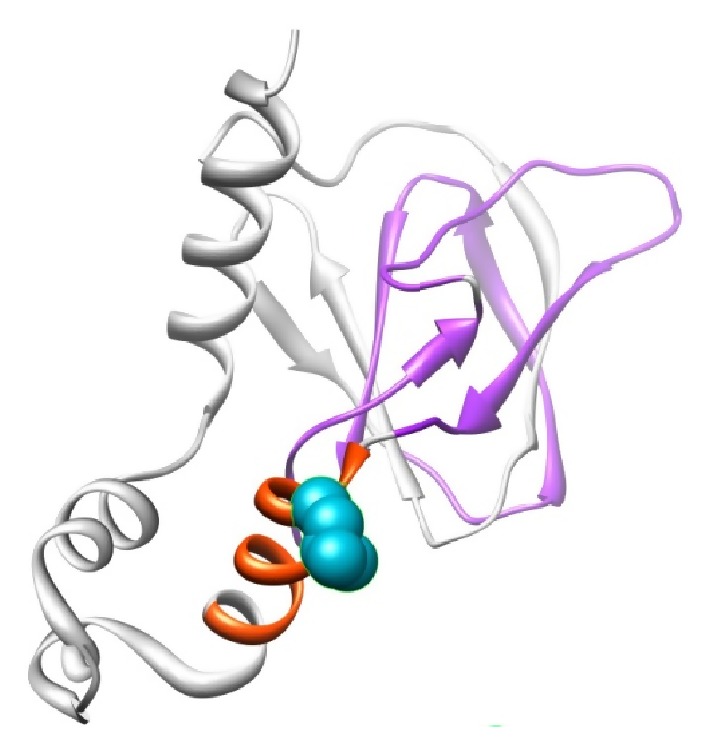
Structural visualization of VHL protein with PDB ID: 1LM8 shown in ribbon representation. The protein is colored in orange [157–166: interaction with Elongin BC complex] and violet [100–155: involved in binding to CCT complex] color; the side chain of the native residue is colored cyan and shown as small balls.

To investigate the structural and dynamical information about the native and mutant proteins at the atomic level, molecular dynamics simulation was performed. It was carried out for 25 ns using GROMACS 4.6 package [21]. To create the mutant model of pVHL, we downloaded X-ray crystallized structure 1LM8 with 1.85 Å [22] from the Protein Data Bank [23]. Mutation analysis was performed using SWISSPDB viewer at position 158 [24]. Human pVHL contains two domains, alpha and beta domain. In our analysis we used “V” chain of PDB ID 1LM8 (Figure 2). The UCSF Chimera (http://www.cgl.ucsf.edu/chimera/) was used to visualize the protein models. Then the entire molecular system was subjected to energy minimization by steepest-descent followed by conjugated gradient method implementing GROMOS96 43a1 force field [25]. Energy minimized structures of native and mutant protein molecules were used as a starting point for MD simulations. We solvated native and mutant proteins in a cubic box with simple point charge (SPC) water molecules [26]. Periodic boundary conditions were applied on the system to maintain the number of particles and constant pressure and temperature. Berendsen temperature coupling method [27] was used to regulate the temperature inside the box. Isotropic pressure coupling was performed using Parrinello-Rahman method. In order to obtain electrically neutralized system, we utilized GENION from the GROMACS package to replace random water molecule with Na^+^ or Cl^−^ ions. In our analysis, we added six chloride ions [Cl^−^] in the simulation box to neutralize the system. Position restrained molecular dynamics simulation was performed at 300 K for 25000 ps to equilibrate the system in an aqueous environment. The temperature was kept constant by using Berendsen algorithm with a coupling time of 0.2. To constrain bond lengths involving hydrogen bond formations, the SHAKE algorithm [28] was used at a time step of 2 fs. The coordinates were saved at regular time intervals of 1 ps. The coulomb interactions were truncated at 0.9 nm, and the Van der Waals force was maintained at 1.4 nm.

## 3. Results

**Figure 3:**
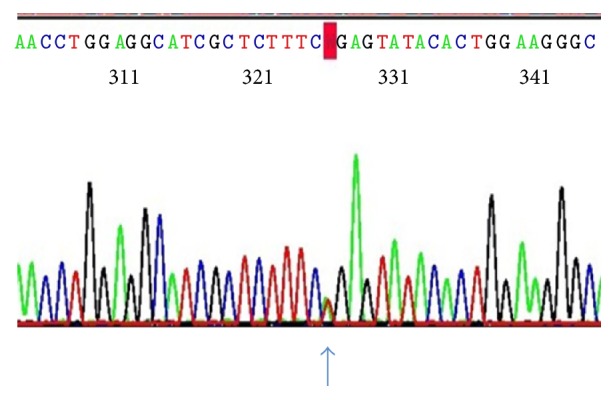
Electropherogram showing the heterozygous mutation* c.473T>A* in exon 3.

Sequence analysis showed a novel heterozygous single nucleotide base substitution in the two affected individuals, which was not seen in the unaffected individual. The variant was seen in* exon 3*,* c.473T>A* [*TRANSCRIPT ID- ENST00000256474, NM_000551*] (Figure 3), with genomic coordinate chr3: 10,149,796 [*hg38 build*] leading to substitution of amino acid leucine to glutamine at position 158 [p.L158Q] in the Elongin C binding domain of pVHL. After a thorough search of databases and literature, this variation was previously unreported as a causative variant of VHL. However, according to the dbSNP build 142, this variant has been flagged as clinically associated, the details of which will be scrutinized further under Section 4. Deletion analysis did not reveal any major loss of genetic material in all the three samples. This variant was also not observed in 50 age and ethnicity matched controls. Further in the two affected individuals a variation in* intron 1*,* c.340+5G>C*, with genomic coordinate 10,42,192 [*hg38 build*] was also observed. This has been previously reported as a benign variant [29].

### 3.1. *In Silico* Analysis

So far among VHL patients the amino acid change at position 158 from leucine to glutamine has not been reported previously. In view of this being a new variant, we considered doing an extensive* in silico* analysis. The results of the eight different* in silico* methods SIFT, PolyPhen 2, SNAP, PROVEAN, MUpro, iStable, MuStab, and I-Mutant Suite to predict the impact of p.L158Q are shown in Table 1. Sequence based methods SIFT and PROVEAN classified the variant as deleterious and structure based methods PolyPhen 2 and SNAP classified the variant as possibly damaging and nonneutral, respectively. In order to quantify the destabilization effects, the protein stability change upon mutation was evaluated by calculating the difference in folding free energy change between native and mutant proteins (DDG or ddG). The results from protein stability predictors, namely, iStable, MUpro, MuStab, and I-Mutant Suite, indicated that the p.L158Q is less stable and deleterious (Table 1). The mutation analysis of amino acid at their corresponding position 158 was performed by SWISS-PDB viewer independently to achieve modeled structures.

### 3.2. Effect of Mutation on Protein Structure

**Figure 4:**
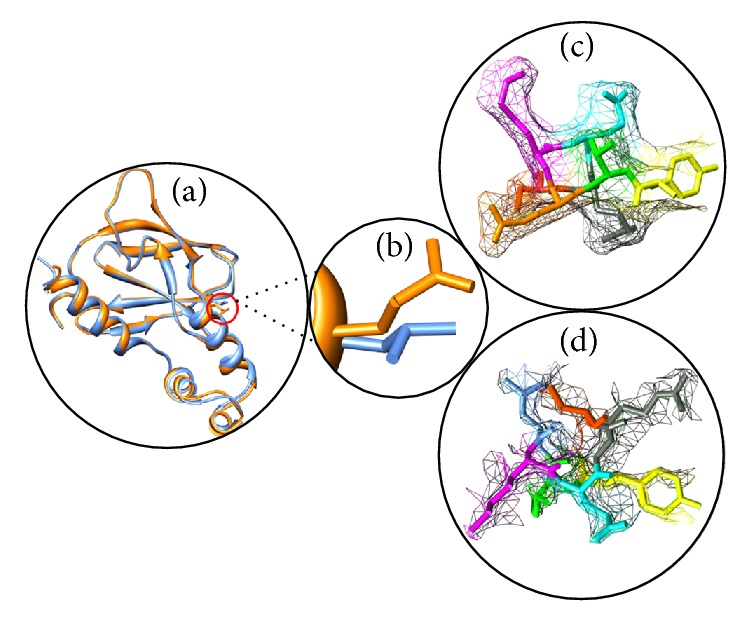
(a) Superimposed structure of native and mutant structure of VHL. (b) Close-up view of native amino acid leucine (orange) and mutant amino acid glutamine (blue) in dot. (c) Local environment change visualized for native amino acid [leucine] in dot model. (d) Local environment change visualized for mutant amino acid [glutamine] in dot model. These figures were drawn using Chimera.

Mutation leads to change in amino acid properties which in turn lead to loss or gain of interactions and hydrogen bonds. This may disrupt the local environment based on the structural interactions in the nearby sites of substitution and manifest their deleterious effects by bringing in changes in structural characteristics such as change in size, surface charge distribution, and hydrophobic contacts [19]. Local environment change upon mutation was defined using PyMol within 4A and visualized using UCSF Chimera (http://www.cgl.ucsf.edu/chimera/). No local environment change was observed upon substitution of leucine by glutamine at the 158th position in VHL (Figure 4). We defined the local environment of native and mutant amino acid by HOPE server to portray the distinct pattern of amino acid substitutions on protein structure based on size, charge, and hydrophobicity. The mutant residue is much bigger than the wild-type residue. The hydrophobicity of the wild-type and mutant residue differed; mutant residue is more hydrophobic than the wild type. The mutation might cause loss of hydrophobic interactions with other molecules on the surface of the protein [18].

### 3.3. Conservation Analysis

**Figure 5:**
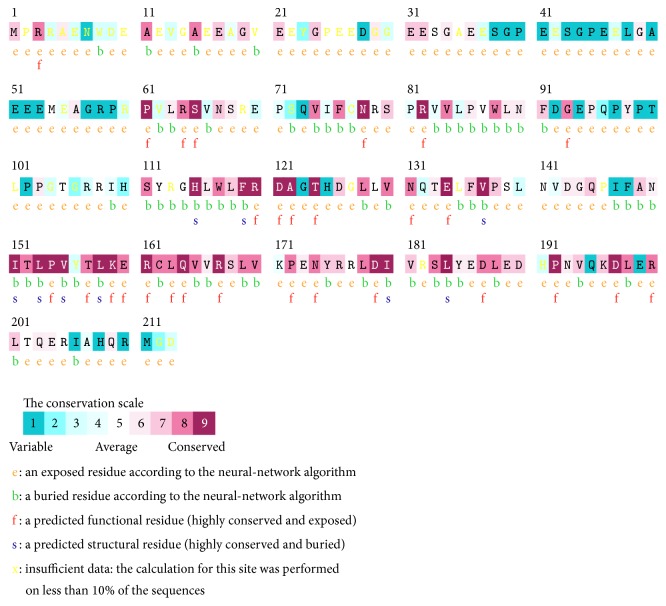
The conservation pattern of amino acid sequence in VHL. The location of amino acid residues in VHL based on the evolutionary conservation pattern. Color intensity increases with a degree of conservation (variable (1–4), intermediate (5-6), and conserved (7–9)).

Primary sequence of a protein provides the most direct information regarding the clues for functional mutation sites. Multiple sequence alignments of amino acid residues provide valuable information about the conservation pattern and represent localized evolution. The substitutions of conserved residues are mostly deleterious in nature. In this context, we applied ConSurf to predict the evolutionary significance of the missense variant p.L158Q. Bayesian analyzer ConSurf predicted the p.L158Q as highly conserved (Figure 5). The obtained results are in agreement with the* in silico* prediction tools that predicted p.L158Q as highly deleterious and less stable.

### 3.4. Molecular Dynamics Simulation Analysis

In order to measure the effect and conformational change upon mutation, we conducted molecular dynamics simulation for 25 ns. Structural properties of the native and mutant protein were calculated from the trajectory files with the built-in functions of GROMACS software. The trajectory files were analyzed through the verity of GROMACS utility. To generate the 3D backbone of the native and mutant protein, RMSD, RMSF, hydrogen bonding, and SASA analysis were plotted for the simulations using Graphing, Advanced Computation and Exploration (GRACE) program.

**Figure 6:**
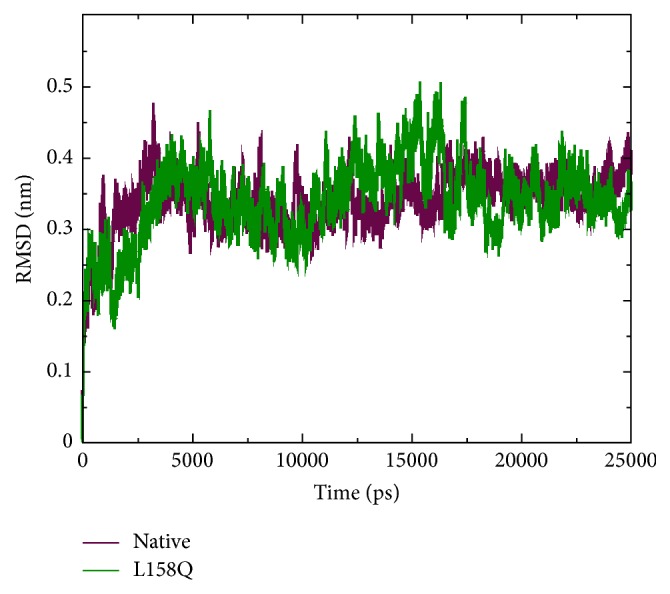
Backbone Root Mean Square Deviation (RMSD) of native and mutant (L158Q) VHL protein. The ordinate is RMSD (nm) and the abscissa is time (ps).

RMSD for all the backbone atoms from the initial structure as a function of time was calculated for the trajectories of the native and mutant protein. In 25 ns trajectory files of RMSD (Figure 6), we can visualize the change in deviation between the native and mutant protein which illustrates the mild or no stabilizing effect on the structure. Mutant protein trajectory showed slightly distinct fashion of deviation after ~12 ns when compared to native, followed by smaller deviations for the rest of the simulations. The RMSDs trajectories of both the simulations reach a stable value after the relaxation period of ~5 ns led to the conclusion that the simulation produced stable trajectories, which provides a suitable basis for further analysis.

**Figure 7:**
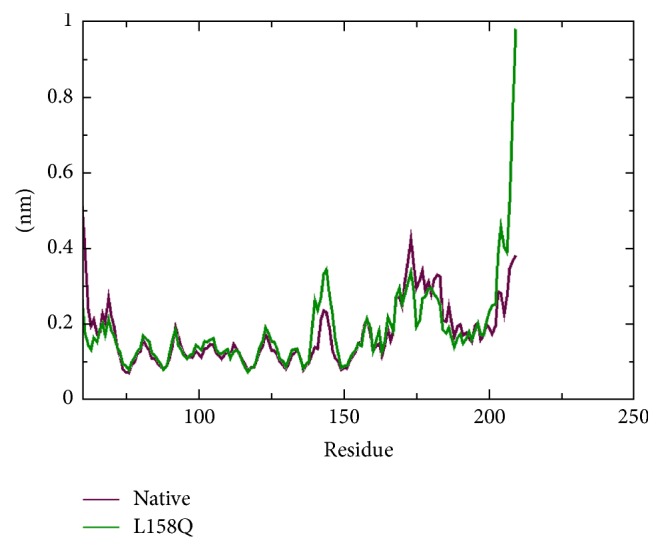
C-alpha Root Mean Square Fluctuation (RMSF) of native and mutant (L158Q) VHL protein. The ordinate is RMSF (nm) and the abscissa is amino acid residues.

**Figure 8:**
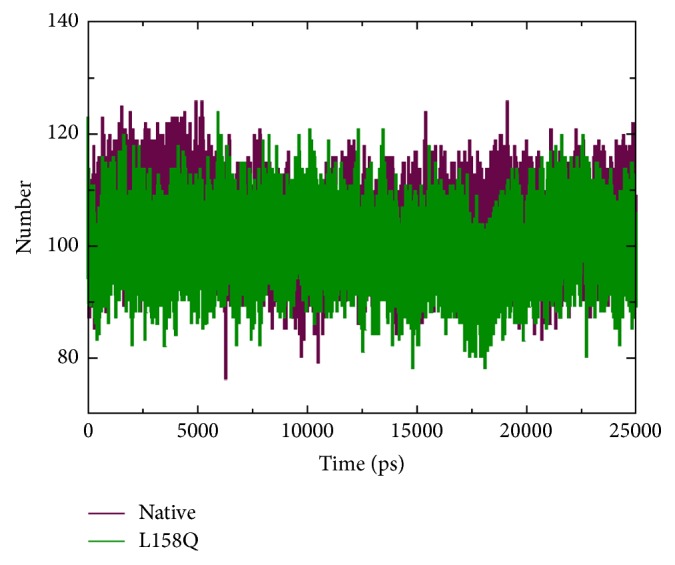
Total number of hydrogen bonds formed between native and mutant (L158Q) VHL protein.

**Figure 9:**
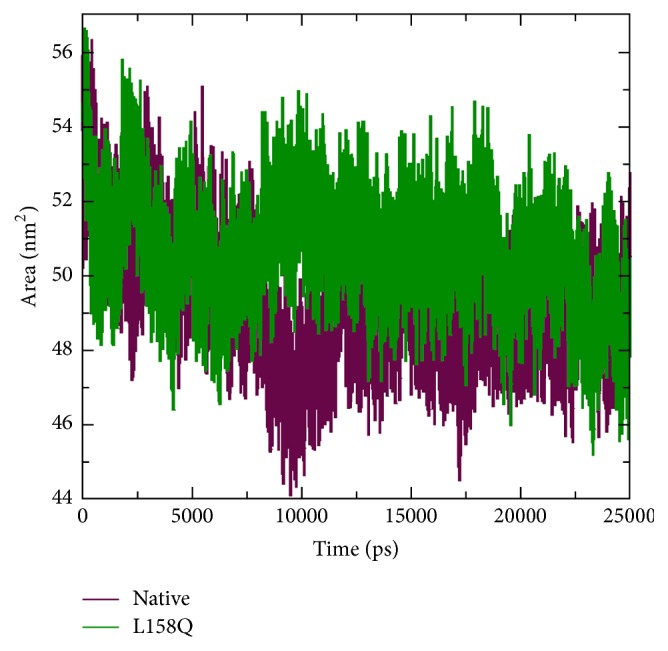
Solvent accessible surface area (SASA) analysis of native and mutant (L158Q) VHL protein.

The change in conformational flexibility of the native and mutant protein was calculated using RMSF values of C*α* atom (Figure 7). Analysis of fluctuation value revealed the presence of higher degree of flexibility in the mutant structure as compared to the native protein. The two proteins display a different pattern of fluctuation in few regions depicting that the mutation affected the flexibility of protein. Moreover, it was very interesting to observe that the amino acid residues present in the region of Elongin C binding of pVHL showed very high fluctuation. This illustrates that the mutation has affected the conformation of protein in a different fashion, which caused the rise in flexibility of residues whereas it induced constraints in the flexibility of the residues in other regions. We conclude from these findings that the mutation induces increased fluctuations in some parts of the structure while not decreasing the overall stability of this structure as judged by the RMSD. Hydrogen bond accounts for a major factor for maintaining the stable conformation of protein. Hydrogen bond analyses of native and mutant proteins were performed with respect to time to understand the relationship between flexibility and hydrogen bond formation. Notably mutant structure showed significantly less number of hydrogen bonds formation during the simulation as compared to the native structure (Figure 8). Atomic flexibility is greatly dependent on the number of hydrogen bonds formed by amino acids. Solvent accessible surface area (SASA) of a biomolecule is that it is accessible to the solvent and it can be related to the hydrophobic core. Solvent accessibility was divided predominantly into buried and exposed region, indicating the least accessibility and high accessibility of the amino acid residues to the solvent. Solvent accessible area was calculated for native and mutant trajectories values and depicted in Figure 9. It is evident that native and mutant exhibited similar fashion of solvent accessible surface area of ~48–56 nm^2^ in the simulation period of ~0–7 ns and very low solvent accessible area of ~46 nm^2^ in the simulation period of ~8-9 ns (Figure 9). Increase or decrease in the solvent accessible surface area indicates change in exposed amino acid residues and it could affect the tertiary structure of the proteins.

## 4. Discussion

**Figure 10:**
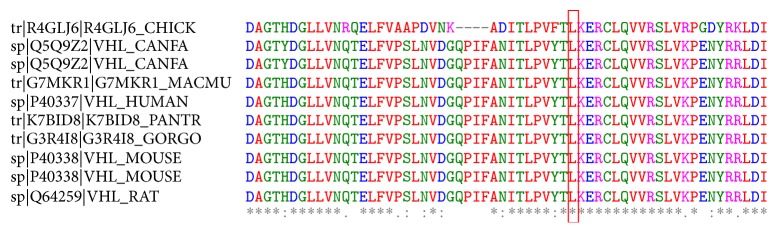
Evolutionary conserved pattern of leucine residue at the 158th position in VHL across cross species. Multiple sequence alignment was performed by MUSCLE.

The variant observed in our study is heterozygous, in keeping with the autosomal dominant pattern of inheritance of VHL. The variant in exon 3* c.473T>A* identified in our patients results in a leucine-to-glutamine substitution at amino acid 158 in the Elongin C binding domain of pVHL. For the regulation of hypoxic gene response pathway, it is essential that pVHL forms a complex with Elongin C and Elongin B. Elongin C directly binds to pVHL whereas Elongin B binds to Elongin C thereby stabilizing the complex [30]. This complex in turn recruits CUL2 and regulates ubiquitination of proteins involved in hypoxic gene response pathway.* In vitro* protein binding studies have demonstrated that residues from 157 to 171 on pVHL form the Elongin C binding site [31]. This region [157–171] forms a helix in the crystal structure that fits into the concave surface present on Elongin C and has been a hot spot for mutations causing VHL. The protein binding studies have also revealed that residues at T157, L158, R161, and C162 are crucial for Elongin C binding [32]. Of these residues L158 produces the strongest of contacts with Elongin C as per* in vitro* alanine scanning experiments [substitution of leucine at position 158 by alanine]. Along with C162 and R161, L158 forms the most significant Van der Waals contacts, with Elongin C residues 17–50.* In vitro* alanine scanning experiments have clearly demonstrated the complete inactivation of pVHL leading to absolute loss of Elongin C binding when leucine at position 158 is replaced by alanine [31]. In addition as Figure 10 illustrates leucine residue at this position is highly evolutionarily conserved making it functionally a crucial amino acid for pVHL. ConSurf analysis provided the highest score of 9 in the conservation scale for the residue L158 as well revealing buried nature of the residue. Previously leucine-to-proline [p.L158P] and leucine-to-valine [p.L158V] change at this position have been reported as causative variants of VHL disease [32]. These evidences suggest the importance of residue leucine at position 158.

To estimate whether the amino acid substitution L158Q in our proband can affect protein function we applied web based programs SIFT, PolyPhen 2, SNAP, PROVEAN, iStable, MUpro, MuStab, and I-Mutant Suite.* In silico* prediction analysis revealed the p.L158Q as deleterious and less stable. Analytical tool HOPE predicted protein structural alterations due to heavier and more hydrophobic mutant residue, also predicting possible loss of hydrophobic interactions at the surface of the protein. Molecular dynamics simulation analysis showed formation of less number of hydrogen bonds and altered flexibility of molecules in and around the region due to the mutation, affecting ultimately the final tertiary structure of pVHL. Summating the results of* in silico* analysis, it can be reliably concluded that p.L158Q is not a neutral variant.

As mentioned earlier in Section 3 according to the dbSNP build 142,* c.473T>A* with genomic coordinate 10,149,796 [*hg38 build*] has been considered a flagged SNP [single nucleotide polymorphism] with unknown minor allele frequency [MAF] and clinically associated. The SNP was deemed clinically associated based on the locus specific database by a single submitter in a somatic sample with germline allele being T. The frequency of allele A is unknown [allele A observed only once in a somatic sample], which was not validated and has never been reported in 1000 genomes or in any other source like HGMD.

Further, the change was not observed in any of our 50 age and ethnicity matched controls, making* c.473T>A* being a polymorphism highly unlikely. The two single nucleotide base substitution variants were shown to segregate with VHL phenotype in this family in which the variant in intron 1, c.340+5G>C, is previously reported and is a recognized neutral variant. Therefore it is possible that the variant in exon 3* c.473T>A*, a missense variant [p.L158Q] with unknown MAF, never reported previously in any database of germline variants, which segregated with VHL phenotype only in the family and which was not observed in the ethnic population the proband belongs to, maybe is disease causing.

### 4.1. Genotype-Phenotype Correlation

In the past it was generally believed that protein truncating mutations are considered to be responsible for many of the Type 1 VHL cases; however growing evidence indicates that it is actually complete loss of function of pVHL irrespective of the type of the mutation that leads to Type 1 VHL. Missense mutations in Elongin C binding domain have been established as a cause of Type 1 VHL by involving key amino acids leading to complete loss of function of pVHL as described earlier. This is in keeping with the phenotype observed in the proband and his family. This also highlights the role of an independent pathway other than hypoxic gene response pathway for the genesis of pheochromocytoma [33]. Usually in pheochromocytoma the missense variations which account for 80–95% [34] of mutations in Type 2 VHL leave pVHL with some residual activity [2] which is in contrast to the possibility of complete abrogation of Elongin C binding in our case. This could explain the absence of pheochromocytoma in the proband's family. However transethnic differences in genotype-phenotype correlations are described. As an example p.L158V which was associated with Type 1 VHL in Caucasians was associated with Type 2 disease in Japanese [35]. Hence even with the entire available database on mutations and basic research, phenotype prediction is difficult.

### 4.2. Biological Relevance

It is clear from the alanine scanning experiments and previous literature that the mutations that affect L158 lead to complete abolishment of Elongin C binding and thereby complete inactivation of pVHL. The highly conserved amino acid leucine at position 158 forms one of the strongest bonding regions with Elongin C. Hence mutations of this amino acid affect interactions with Elongin C and in turn a host of other molecules. This paper illustrates in general the importance of Elongin C binding domain and L158 in particular in the functioning of pVHL.

## 5. Conclusions

*In silico* predictive analyses, available relevant literature, and results of genotype analysis of healthy controls provide strong evidence for pathogenicity of the previously unreported variant* c.473T>A* [*p.L158Q*]. Hence, this variant is highly likely to be causative of VHL. Our finding supports the functional importance of residue L158 in pVHL functioning and further illustrates the diversity of* VHL* gene defects underlying phenotypic heterogeneity in VHL. The results of this study can serve as a harbinger of functional studies to be performed in the future. The analysis also helped us in genetic counseling, as we could reassure the proband about his son's status.

## Figures and Tables

**Table 1:** Summary of prediction scores of *in silico* methods.

*In silico* methods	Prediction
SIFT	Deleterious
PROVEAN	Deleterious
PolyPhen 2	Probably damaging
SNAP	Nonneutral
iStable	Decrease protein stability
MUpro (DDG)	Decrease protein stability
MuStab (DDG)	Decrease protein stability
I-Mutant Suite (DDG)	Decrease protein stability
